# The Transcriptome Profiling of Flavonoids and Bibenzyls Reveals Medicinal Importance of Rare Orchid *Arundina graminifolia*

**DOI:** 10.3389/fpls.2022.923000

**Published:** 2022-06-23

**Authors:** Sagheer Ahmad, Jie Gao, Yonglu Wei, Chuqiao Lu, Genfa Zhu, Fengxi Yang

**Affiliations:** ^1^Guangdong Key Laboratory of Ornamental Plant Germplasm Innovation and Utilization, Environmental Horticulture Research Institute, Guangdong Academy of Agricultural Sciences, Guangzhou, China; ^2^Guangdong Laboratory for Lingnan Modern Agriculture, Guangzhou, China

**Keywords:** *Arundina graminifolia*, medicinal constituents, model orchid, HPLC-MS/MS, WGCNA

## Abstract

Orchids are very important flowering plants that spend long juvenile phases before flowering. Along with aesthetic importance, they are rich sources of medicinal components. However, their long reproductive cycle is the major hurdle to study the medicinal efficacy. *Arundina graminifolia* is a rare orchid that grows fast, unlike other orchids, and this characteristic makes it an ideal plant to study the medicinal enrichment of orchids. Therefore, this study presents the identification of important medicinal components in various parts of *A. graminifolia*. Transcriptome analysis was performed for five stages (FD1–FD5) of flower development and four tissue types (mature flower, silique, root, and leaf) to ascertain genetic regulators of flavonoids and bibenzyls. Most of the genes showed the highest expression in roots as compared with other tissues. Weighted gene coexpression network analysis (WGCNA) was performed to identify the coexpression modules and the candidate genes involving biosynthesis pathways of these chemicals. MEyellow module contained the highly coexpressed genes. Moreover, the concentrations of phenylpropanoid, bibenzyls, and flavone were ascertained through high-performance liquid chromatography-tandem mass spectrometry (HPLC-MS/MS). Phenylpropanoid and bibenzyl were comparatively high in the leaf, while flavone showed a high concentration in the stem. The selected candidate genes [bibenzyl biosynthesis (BIBSY212), CYP84A1, CYP73A4, 4CLL7, UGT88B1, UGT73C3, anthocyanin synthase (ANS), phenylalanine ammonia-lyase (PAL), flavanone synthase FLS, and CHS8] were validated through quantitative real-time PCR (qRT-PCR). Most of these genes showed high expression in leaf and root as compared with other tissue. Therefore, the presence of bibenzyls and flavonoids in different parts of *A. graminifolia* and their molecular regulators can provide a quick source to decipher the medicinal efficacy of orchids.

## Introduction

The Orchidaceae family is one of the largest angiosperm families and mainly contains ornamental flowers (Cai et al., 2015b; Wong et al., 2017). About 100,000 million species are grown worldwide, showing the immense horticultural importance of orchids (Ahmad et al., 2021b). Orchids, such as *Cymbidium* and *Phalaenopsis*, bloom after a long vegetative phase of 2–3 years (Ahmad et al., 2021a). However, a rare orchid *Arundina graminifolia*, completes its vegetative phase in 6 months and then continues flowering (Ahmad et al., 2021a,b, 2022). It is commonly called bamboo orchid due to its shape similar to bamboo plant. Its significantly short vegetative phase makes it an ideal plant to study different aspects and benefits of orchids; especially the medicinal components. Although a few reports describe the identification of different chemicals in bamboo orchids, the molecular regulation remains unattended. Revealing the genetic regulation of flavonoids and bibenzyls in *A. graminifolia* can serve as a useful source to extend the knowledge to chemical identification in other orchids.

*Arundina graminifolia* is used as a medicinal plant in China because of the presence of flavonoids, stilbenoid, and phenols in its extracts, which exhibit antioxidant, anti-virus, anti-tumor, and other medicinal properties (Ai et al., 2019). The whole plant is a famous Dai medicine in China, curing food poisoning, blood stasis, and liver toxicity (Liu et al., 2007; Zhang et al., 2012; Xiaohua et al., 2015). In India, it is used as an emollient and antibacterial agent (Panda and Mandal, 2013). In Bangladesh, it used to cure rheumatism (Hossain, 2009). Previous studies have reported stilbenoids as the major secondary metabolites in bamboo orchid, revealing the structural diversity of phenanthrenes (Liu et al., 2005), diphenylethylenes (Hu et al., 2013b; Li et al., 2013a; Gao et al., 2014; Meng et al., 2014; Yang et al., 2014), bibenzyls (Majumder and Ghosal, 1993; Du et al., 2014), and other phenolic compounds (Gao et al., 2012; Hu et al., 2013a; Lidan et al., 2013; Li et al., 2013b; Niu et al., 2013). Bibenzyls are effective antitumor agents due to their antioxidant and cell-protective properties (Gong, 2003; Zhang et al., 2007; Barbosa et al., 2009; Li et al., 2010; Su, 2011; Cai et al., 2015a). Moreover, Bibenzyls are used in several drugs and skincare products (Zhang et al., 2007; Hossain, 2011).

In *Dendrobium officinale*, the active medicinal constituents include alkaloids, terpenes, polysaccharides, flavonoids, phenols, and bibenzyl (Zhang et al., 2016; Tang et al., 2017). Bibenzyls have also been identified in *Dendrobium sinense* (Chen et al., 2014). In the orchid *Epidendrum rigidum*, bibenzyls showed phytotoxic activity, suggesting that orchid bibenzyls can be a good lead for developing novel herbicides (Hernández-Romero et al., 2005). The biosynthesis of bibenzyls is regulated by four key enzymes. The biosynthesis of dihydro-m-coumaroyl-CoA begins with phenylalanine and ends with a cinnamate molecule catalyzed by phenylalanine ammonia-lyase (PAL). The cinnamate is incorporated into m-coumaric-CoA with the catalyzation of cinnamate 4-hydroxylase (C4H). Then, dihydro-p-coumaroyl-CoA is produced from p-coumaric-CoA with the catalyzation of 4-coumarate: CoA ligase (4CL). Dihydro-m-coumaric acid is synthesized at the same time from m-coumaric acid with the incorporation of cytochrome P450 (CYP450) (Majumder et al., 2001; Peled-Zehavi et al., 2015; Jeong et al., 2016; Ibdah et al., 2017; Yahyaa et al., 2017).

Cytochrome P450 genes are the important regulators of secondary metabolites and bibenzyls (Adejobi et al., 2021). They play a role in regulating the production of defense-related secondary metabolites (Gomez et al., 2011). The expression of CYP450s significantly affects the quality of bibenzyls in plants. The pathway for secondary metabolite biosynthesis involves different regulatory modifications and physiological factors in response to environmental changes. Usually, the secondary metabolites, such as bibenzyl, accumulate in low concentrations in the tissues (Hussain et al., 2012). However, the market demands high levels of bibenzyl accumulation in plant tissues to facilitate the drug-making. Therefore, it is an immediate need to dissect the molecular mechanisms underlying the biosynthesis of bibenzyls in *A. graminifolia*, which can provide continuous supply of medicinal ingredients much faster than other orchids. The identification of regulatory factors and rate-limiting enzymes, responsible for bibenzyl biosynthesis, is also an area of further exploration in orchids. There is an urgent need of plant-derived sustainable sources of bibenzyl, and *A. graminifolia* has excellent potential to provide a continuous supply of bibenzyl and other medicinal ingredients. Researchers have so far focused on the identification of the chemical components, pharmacological activities, and bioactive substances in *A. graminifolia*. However, the underlying molecular regulation is not discussed.

With the rapid development of RNA-seq technology, transcriptomic data mining offers a great opportunity to discover pivotal genetic regulators or rate-limiting enzymes that control the secondary metabolites in plants (Jia et al., 2015; Zhu et al., 2016). The transcriptome analyses have identified several rate-limiting genes regulating the biosynthesis of lignin in *Apium graveolens* (Pandey et al., 2018), terpenoids in *Eugenia uniflora* (Kumar et al., 2016), and flavonoids in *Solanum viarum, Dracaena cambodiana*, and *Phyllanthus embica* (Meng et al., 2016; Lei et al., 2018; Yuan et al., 2018). However, the biosynthesis pathway and potential gene regulators of bibenzyl biosynthesis in *Arundina* plants remain elusive.

Therefore, this study investigates the accumulation of flavonoid, phenylpropanoid, and bibenzyl in various tissues of *A. graminifolia*. We performed the transcriptome analysis from five developmental stages and four tissue types to identify the putative genes associated with the biosynthesis of flavonoid, phenylpropanoid, and bibenzyl. The findings provide new resources for the rapid production of medicinal ingredients in orchids.

## Methods

### Plant Materials and Growth Conditions

The bamboo orchid plants were obtained from seeds grown at the greenhouse of the Institute of Environmental Horticulture (Guangdong Academy of Agricultural Sciences, China). The seeds were grown on asymbiotic MS (Murashige and Skoog) media. The seedlings were transferred to a greenhouse and kept at a temperature of 25/20°C day and night, with a 16/8 h of photoperiod. Samples were taken from five stages of flower development (FD), stages 1–5, fully mature flowers, fruits, leaves, and root, as previously mentioned (Ahmad et al., 2021a). Three technical and biological repeats were collected in liquid nitrogen and immediately stored at −180°C for RNA extraction.

### RNA-Seq Library Preparation and Sequencing

RNA was extracted using the TaKaRa kit and cDNA libraries were made for RNA. From total RNA, mRNA was filtered using the Oligotex mRNA Midi Kit (QIAGEN, GERMANY), followed by quality and quantity check using a Nano-Drop 2000 spectrophotometer (Thermo Scientific, USA). The cDNA libraries were made using the Illumina manufacturing protocol (Ahmad et al., 2021a). The total mRNA was subjected to first and second strand cDNA synthesis and adapter ligation, and low cycle enrichment was achieved by the TruSeq^®^RNA HT Sample Prep Kit from Illumina (USA). The products were evaluated with the Agilent 2200 TapeStation and Qubit^®^2.0 of Life Technologies (USA). Dilutions were made to 10 pM for generating *in situ* clusters on HiSeq2500 pair-end flow cell. The 2 × 100 bp sequencing was performed, resulting in 60 M reads per sample. Transcriptomic *de novo* was done with the Trinity program using default parameters (Grabherr et al., 2011).

### Differentially Expressed Gene Analysis

The FPKM (fragments per kilobase of exon model per million reads mapped) expression of genes was calculated using following formula:


$$\begin{array}{lll} \text{FPKM}\; & = & \left[\text{total exon reads/mapped reads}\;(\text{millions})\right]\\ & & \times \text{exon length}\;(\text{kb}) \end{array}$$

The edgeR package was used to identify significant differences among genes (Ahmad et al., 2022). A threshold level of gene significant difference was calculated at a false discover rate (FDR) of <0.05 and a | log2 ratio| > 1 (2-fold change). The genes were mapped to public annotation databases, such as Kyoto encyclopedia of genes and genomes (KEGG), gene ontology (GO), non-redundant (NR), and KEGG orthology (KO), using the BLASTX program, as previously documented (Ahmad et al., 2021a). The DEGs were also annotated on the UniProt and protein family (PFAM) databases using default parameters. A hypergeometric test was applied using phyper function in R to identify DEGs with enriched terms. Significantly enriched KEGG or GO terms were filtered at *p*-value or *q*-value of ≤0.05.

### Filtering of Phenylpropanoid and Flavonoid Pathway DEGs

The KEGG pathways were searched to find genes related to flavonoids biosynthesis, phenylpropanoid biosynthesis, and flavone biosynthesis. About 500 DEGs were filtered related to these pathways. Selected pathway genes of flavonoid and phenylpropanoid were used to make a heatmap using the pheatmap package of R. The protein–protein interaction analysis was performed at the online String facility using the protein sequences of DEGs as inputs.

### Weighted Gene Coexpression Network Analysis

The weighted gene coexpression network analysis WGCNA package of R was used to perform coexpression and to identify module of coexpressed genes (Ahmad et al., 2021a). Initially, the removal of unqualified genes was performed through the function of goodSamplesGenes. After this, the criterion of scale-free topology was used to select a suitable soft-threshold power using the function of pickSoftThreshold. With the incorporation of one-gene-to-all relationship, the adjacency matrix was converted into topological matrix (TOM) (Yip and Horvath, 2007). TOM-based dissimilarity (1-TOM) function was used to identify genes showing hierarchical clustering. Finally, the modules with highly interconnected gene clusters were created (Ravasz et al., 2002).

### The Quantitative Real-Time PCR Analysis

RNA was extracted from flowers, capsules, leaves, and roots to check the expression of 10 selected candidate genes in the flavonoid and bibenzyl pathway. Total RNA was extracted and cDNA was obtained using the Fermentas protocol. The quantitative real-time PCR (qRT-PCR) was performed in a mixture of 20 μl containing 10 μl SYBR premix Ex-taq™ (Takara, Japan) with the Bio-Rad CFX-96 RealTime PCR system (Bio-Rad, USA). *Actin* was the internal standard used to normalize expression data. The primers are shown in Supplementary Table 3.

### Quantification of Flavonoids and Bibenzyls

The concentrations of flavonoids, bibenzyls, and phenylpropanoids were ascertained for leaf, root, stem, flower, and fruit. The chemical contents were determined following the protocol of high-performance liquid chromatography-tandem mass spectrometry (HPLC-MS/MS) (Aglient), as previously documented (Pan et al., 2010).

### Statistical Analysis

Chemical and qRT-PCR data were analyzed using one-way ANOVA on SPSS software (SPSS Inc., Chicago, IL, USA; ver. 16.0). A significant difference is shown at *p* < 0.05 or *p* < 0.01 level.

## Results

### Transcriptome Analysis and Annotation

The transcriptome analysis produced 71.2 billion high quality reads. Each sample produced about 7.8 billion reads consisting of 10.8–12.8 Gb data (Supplementary Table 1). The data were filtered into 25,353 unigenes and 94,317 transcripts (Supplementary Table 2). The data were annotated to obtain KEGG, GO, Pfam, eggNOG, NR, and SwissProt enrichments. These data have been used to mine genes related to flowering time regulation in our previous researches (Ahmad et al., 2021a,b). However, the abundant data about chemical constituents were not analyzed. Therefore, the DEGs were filtered to obtain genes specific to flavonoids, bibenzyls, and phenylpropanoids pathways.

### Major Enzyme Classes and Pathways

**Figure 1:**
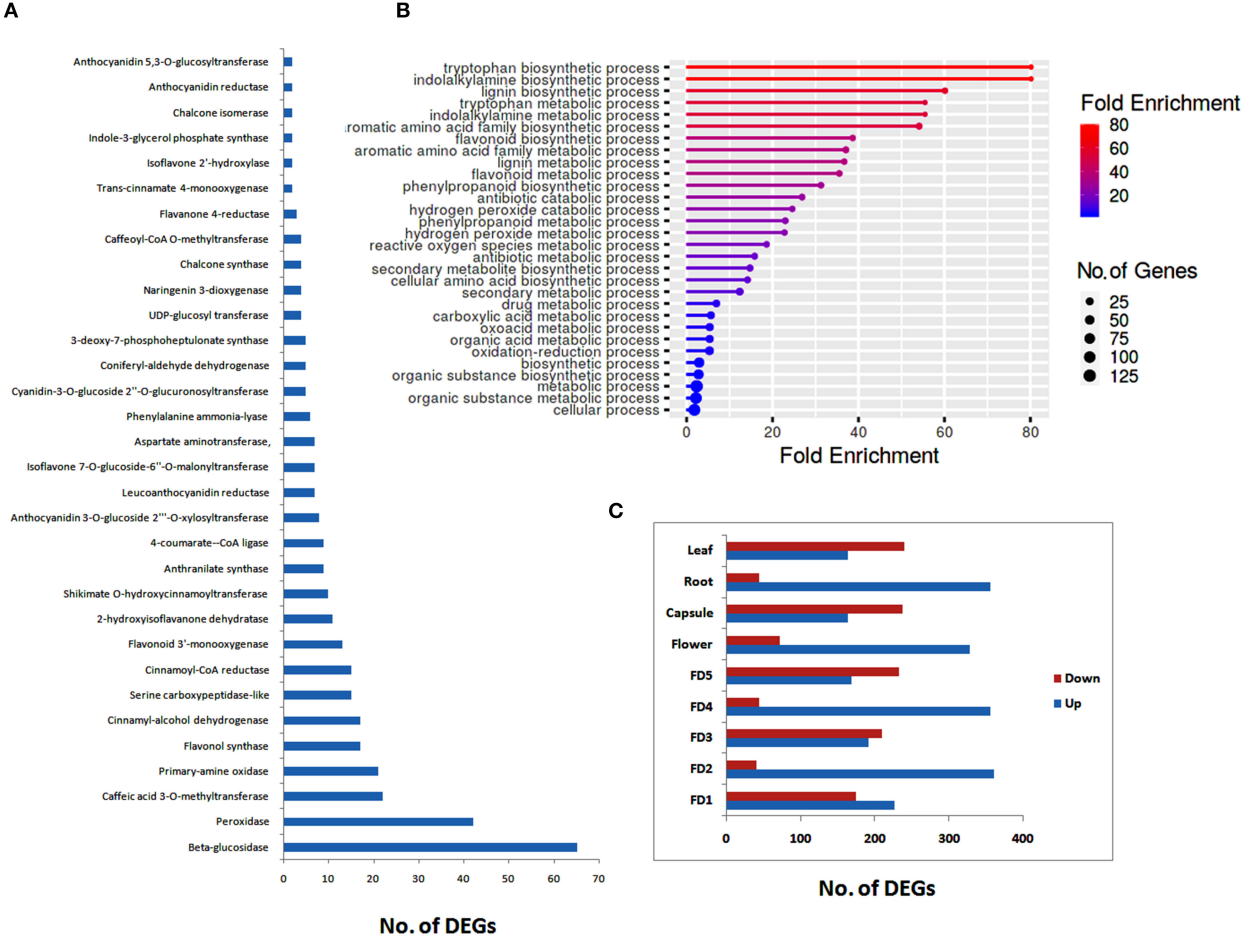
Distribution of secondary metabolite data into enzyme classes **(A)**, major biological process enrichments **(B)** and up- and downregulated differentially expressed genes (DEGs) for different tissues **(C)**.

All the DEGs related to different chemicals were further analyzed to filter different classes of enzymes that may play roles in the regulation of phytochemicals, such as flavonoids and bibenzyls. The highest numbers of genes were related to beta-glucosidases, followed by peroxidases, caffeic acid 3-O-methyltransferases, and primary-amine oxidase (Figure 1A). We identified 17 DEGs related to flavonol synthase, an important enzyme regulating flavonoid biosynthesis. Other major enzymes identified in the biosynthesis pathways of flavonoids and bibenzyls included cinnamoyl-CoA reductase, flavonoid 3'-monooxygenase, 4-coumarate–CoA ligase, UDP-glucosyl transferase, and chalcone synthase (Figure 1A).

Enrichment of different biological processes of DEGs was found using the ShinyGO online enrichment tool. A considerable numbers of DEGs were enriched in flavonoid metabolic process, flavonoid biosynthetic process, phenylpropanoid biosynthetic process, secondary metabolite biosynthetic process, and antibiotic metabolic process (Figure 1B). The highest numbers of genes were related to cellular process and metabolic process.

Stage-specific up- and down-regulated DEGs were also ascertained. The highest numbers of up regulated DEGs were found in root as compared to other stages of flower development or tissue types (Figure 1C). However, in floral development stages, there was no considerable difference between up-regulated and down-regulated DEGs, except for FD2, were a large number of DEGs were up-regulated (Figure 1C).

### Major Regulators of Flavonoids and Phenylpropanoids

**Figure 2:**
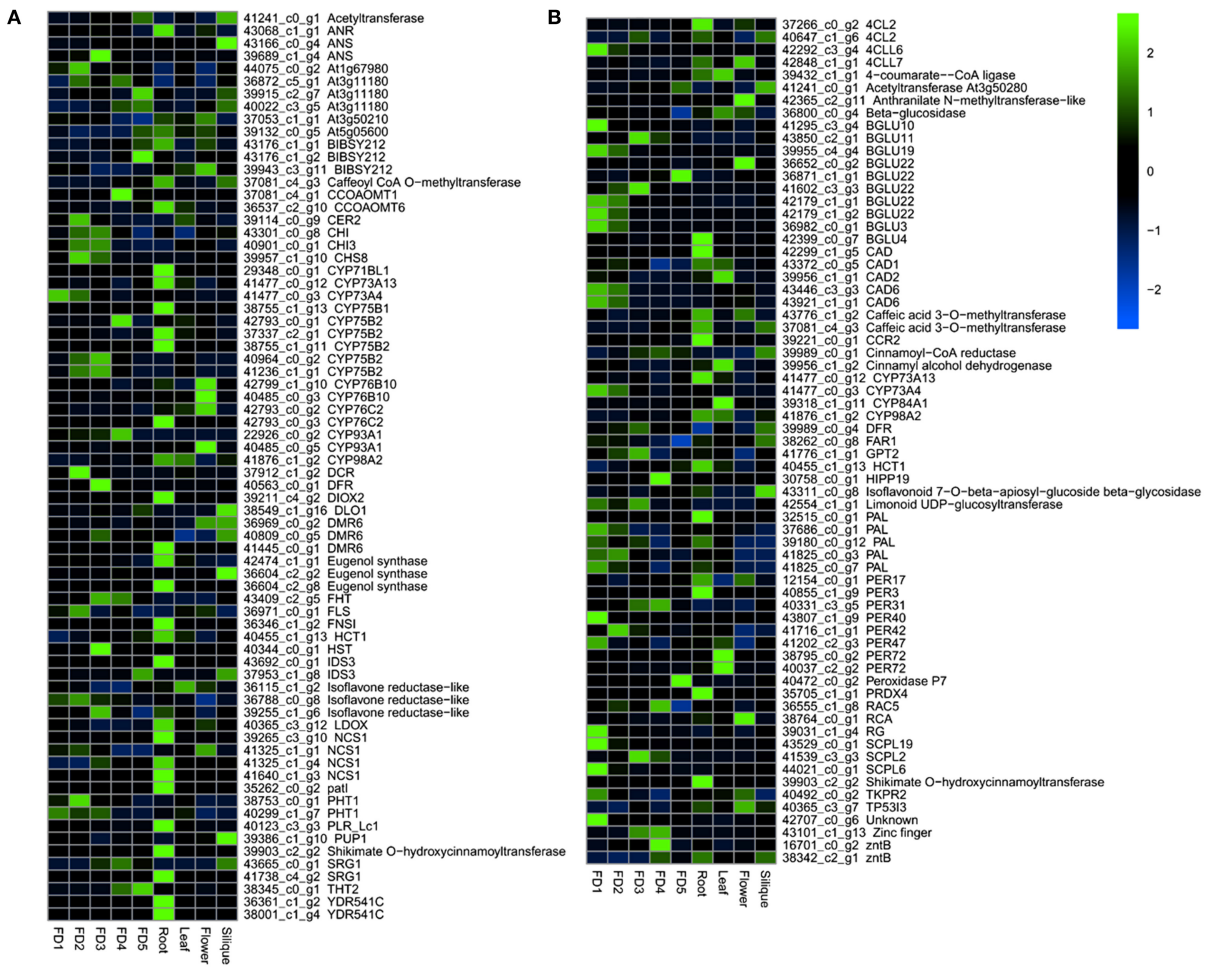
The heatmap of major DEGs involved in the biosynthesis of flavonoids **(A)** and phenylpropanoids **(B)**.

A number of genes were involved in the biosynthetic process of flavonoids and phenylpropanoids (Figure 2). A number of enzymes, such as anthocyanin reductase (ANR), anthocyanin synthase (ANS), bibenzyl synthases (BIBSY), chalcone isomerase (CHI), phytochrome P450 (CYPs), and flavanone synthase (FLS) were identified in the flavonoid and bibenzyl biosynthesis pathways (Figure 2A). In the phenylpropanoid biosynthesis, the major genes identified included 4CLs, BGLUs, CADs, CYPs, PALs, PERs, and SCPLs (Figure 2B).

Most of the flavonoid and bibenzyl biosynthesis pathway genes showed the highest expression in root as compared with other tissues (Figure 2A). The phenylpropanoid pathway genes were highly expressed in the early stages of flower development and root (Figure 2B).

### Root Specificity and Protein–Protein Interaction of Genes

**Figure 3:**
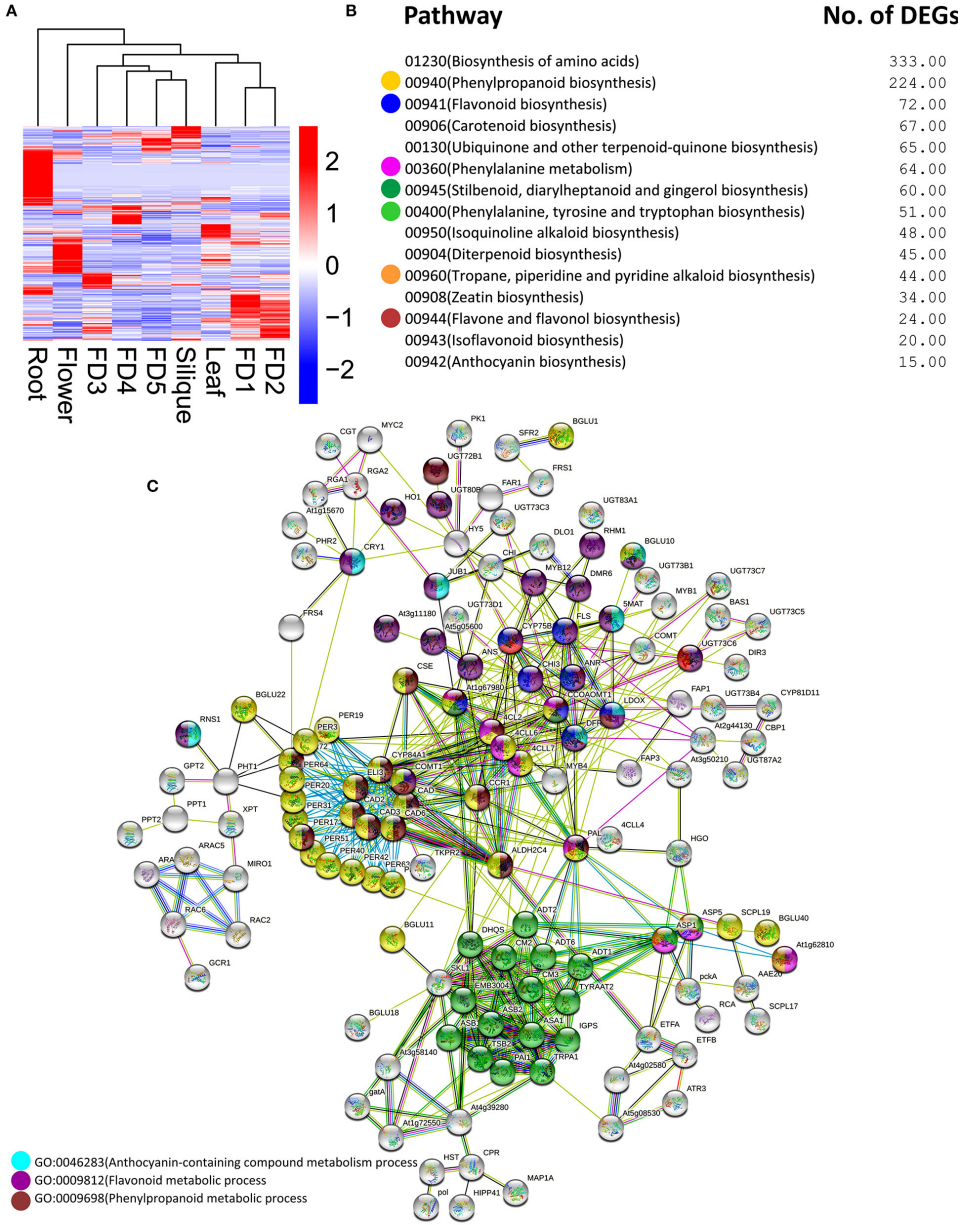
Clustering analysis of DEGs related to secondary metabolites **(A)** major pathways enriched in DEGs related to secondary metabolites **(B)** protein–protein interaction among DEGs related to secondary metabolites **(C)**. The circle colors show the enrichment of pathways shown in part **(B)**.

Plenty of DEGs related to flavonoids, anthocyanins, bibenzyls, terpenoids, and other chemicals, were found in our data. A heatmap clustering of these DEGs showed that most of them were expressed in roots (Figure 3A), which were mostly downregulated in other tissues. In addition, the highest numbers of genes were specifically expressed in root as compared with other tissues. Phenylpropanoid biosynthesis and flavonoid biosynthesis were the abundant pathways along with the biosynthesis of amino acids (Figure 3B). Moreover, the same pathways can be found in the protein–protein interactions among DEGs involving chemical homeostasis (Figure 3C).

### WGCNA Modules in Flower and Non-Reproductive Tissues

**Figure 4:**
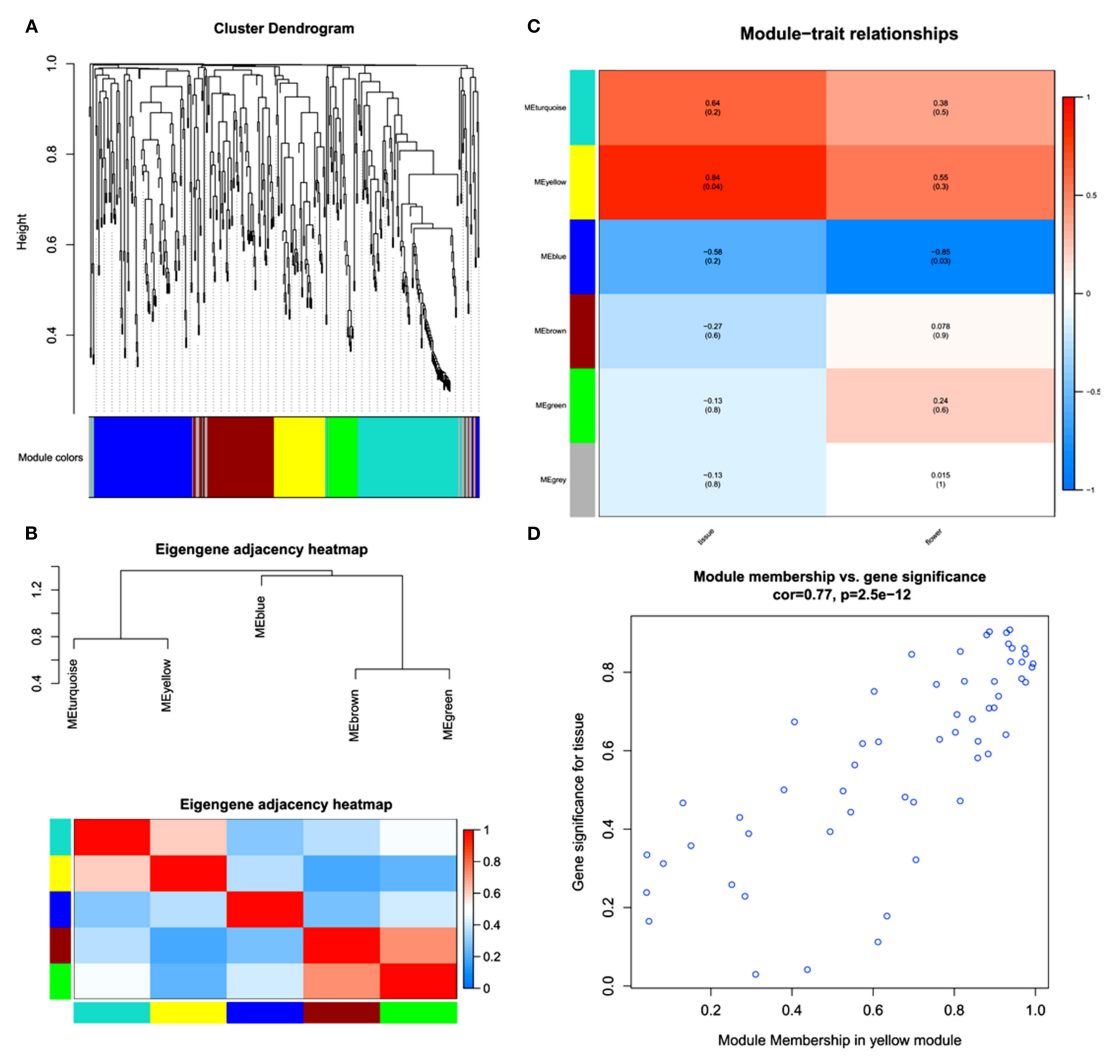
Weighted gene coexpression network analysis (WGCNA); cluster dendrogram **(A)**, eigengene adjacency heatmap of modules **(B)**, key modules with expression intensities **(C)**, and module membership of yellow module for tissue-specific gene significance **(D)**.

A WGCNA was performed to further understand the gene coexpression for phytochemical regulation in *A. graminifolia* (Figure 4). The cluster dendrogram shows the possibility of different modules in the specification of flavonoids and phenylpropanoids (Figure 4A). The modules based on this clustering show two expression sets. The one set contains clusters of upregulated genes, MEturquoise, and MEyellow; while MEblue, MEbrown, and MEgreen contain the clusters of downregulated genes (Figure 4B).

MEyellow showed highly coexpressed genes with greater tissue specificity than flowers (Figure 4C). The MEblue contained the most downregulated set of genes, with more downregulation in flower as compared with tissues. The MEyellow module was further analyzed to know its significance for tissue specificity (Figure 4D). A significant correlation can be seen in the genes for tissues.

### Candidates of Flavonoids and Bibenzyls

**Figure 5:**
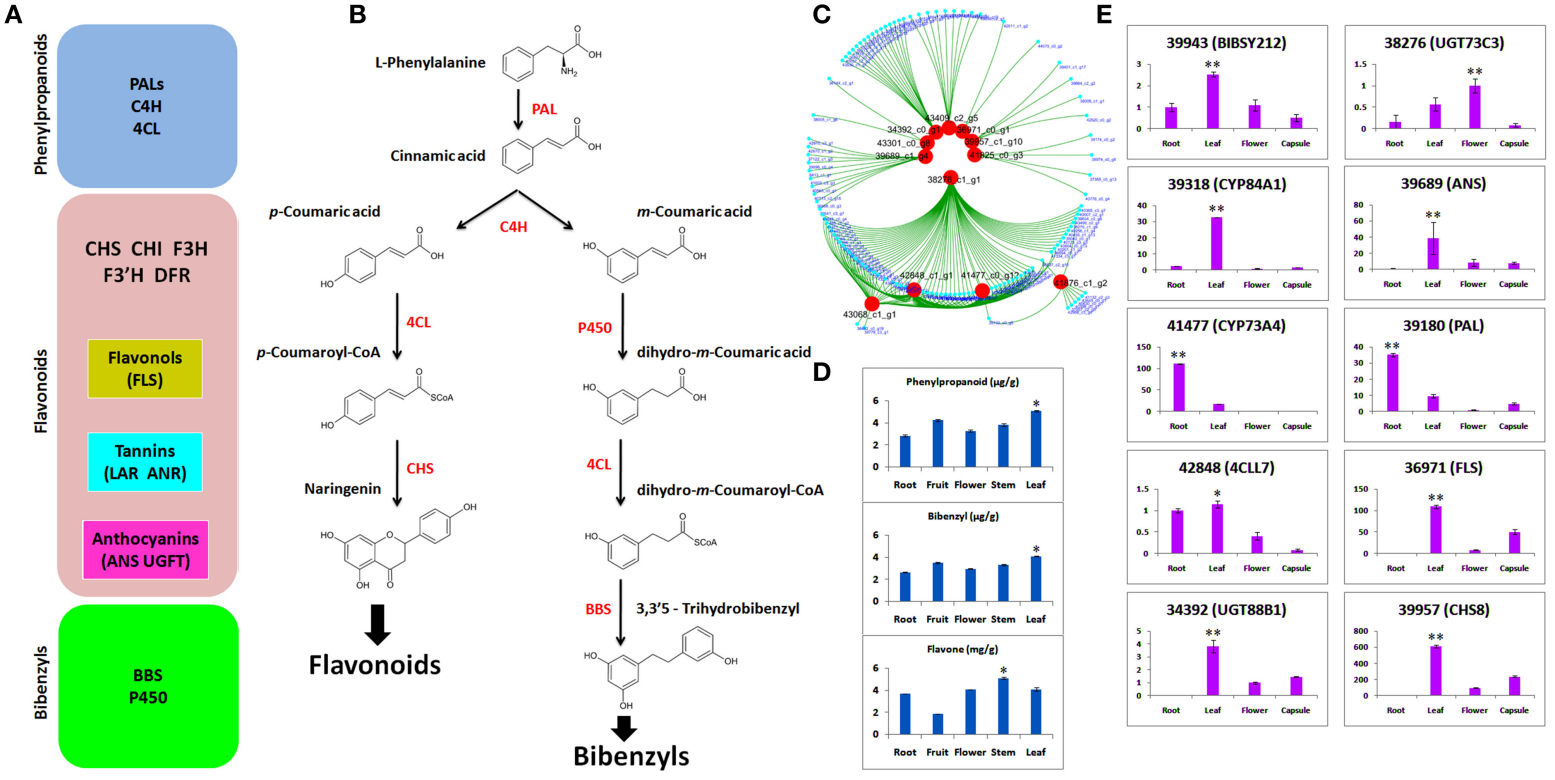
**(A)** Gene regulators of phenylpropanoids, flavonoids, and bibenzyls; **(B)** biosynthesis route of flavonoids and bibenzyls. Colored in red are the enzymes that regulate different steps of biosynthetic pathways; **(C)** selection of candidates that involve biosynthesis of flavonoids and bibenzyls through coexpressed modules; **(D)** concentrations of phenylpropanoids, bibenzyls, and flavonoids through high-performance liquid chromatography-tandem mass spectrometry (HPLC-MS/MS); **(E)** quantitative real-time PCR (qRT-PCR) expression of 10 selected DEGs related to bibenzyl and flavonoid biosynthesis. The statistical significance is shown at *p* ≤ 0.05 (^*^) or *p* ≤ 0.01 (^**^).

We identified DEGs potentially involved in the biosynthesis of phenylpropanoid, flavonoid, and bibenzyls (Figure 5A). Bibenzyl, belonging to sesquiterpenes (Adejobi et al., 2021), is a downstream product of methylerythritol 4-phosphate (MEP) and MVA (mevalone) biosynthesis pathways in plants. Our data also included enzymes for this pathway, such as hydroxymethylglutaryl-CoA synthase (HGMS), mevalonate kinase (MK), 1-deoxy-d-xylulose-5-phosphate reductoisomerase (DXR), 1-deoxy-d-xylulose-5-phosphate synthase (DXS), phosphomevalanote kinase (PMK), 2-C-methyl-d-erythritol 2,4-cyclodiophosphate synthase (MDS), and 4-hydroxy-3-methylbut-2-enyl diphosphate reductase (MDS). L-Phenylalanine is the usual substrate to generate bibenzyl *via* cinnamic acid with the catalysis of PAL (Figure 5B). The C4H catalyzation generates two isomers of m-coumaric acid and p-coumaric acid. Then, using m-coumaric acid as substrate, dihydro-m-Coumaric acid, dihydro-m-Coumaroyl-CoAic, and 3,3'5-Trihydrobibenzyl are synthesized with the catalysis of CYP450, 4CL, and BBS, respectively. We identified two C4H, two PALs, two important CYP450s (CYP84A1 and CYP98A2), three BBS, and two 4CLs (Majumder et al., 2001; Peled-Zehavi et al., 2015; Jeong et al., 2016; Ibdah et al., 2017; Yahyaa et al., 2017).

Based on WGCNA analysis, the clustering of co-expressed modules suggested 12 important genes that are the important regulators of flavonoids and bibenzyls (Figure 5C). These hub genes are the important regulators of bibenzyl and flavonoid biosynthesis pathways shown in Figure 5B.

### Concentration of Major Phytochemicals

Concentrations of phenlypropanoids, bibenzyls, and flavonoids were ascertained through HPLC-MS/MS in five tissues, such as flower, fruit, leaf, stem, and root (Figure 5D). Phenylpropanoid and bibenzyl were abundant in leaf. Flavone is a flavonoid and it was abundant in stem and low in fruit. The higher concentration of bibenzyl was observed in leaf as compared with other tissues.

### The qRT-PCR Validation of Selected Genes

To empirically certify the expressions of DEGs obtained from RNA-seq, we selected 10 important genes responsible for bibenzyl biosynthesis (BIBSY212, 4CCL7, CYP84A1, and CYP73A4) and flavonoid biosynthesis (UGT73C3, PAL, FLS, UGT88B1, and CHS8). The qRT-PCR validation of candidate genes for bibenzyls and flavonoids showed that most of the genes were highly expressed in leaf, as compared with other tissues, few expressed in root and just one expressed in the flower (Figure 5E).

## Discussion

Orchidaceae contains the world's most beautiful flowers with unique shapes, colors, and forms. Potted and cut flower orchids make a huge business (Tokuhara and Mii, 2003; Bhattacharyya et al., 2014). In addition to their economic value, the presence of phytochemicals has been exploited recently for the preparation of vital drugs (Moin et al., 2012; Bhattacharyya et al., 2014). Bioactive compounds, such as polysaccharides, flavonoids, alkaloids, and bibenzyls are integral components of complex processes of drug development (Li et al., 2011; Ng et al., 2012). Polysaccharides possess hepato-protective and immunomodulatory functions, while bibenzyls exhibit anticancer, immunomodulatory, and antioxidant characteristics (Gong et al., 2004; Tang et al., 2017; Li et al., 2018). Several studies have identified genes regulating polysaccharide biosynthesis (He et al., 2015; Zhang et al., 2016; Shen et al., 2017). However, little is documented on the molecular underpinning of bibenzyl biosynthesis in plants, especially in orchis. Bamboo orchid is a highly prized unique orchid and has been playing an important role in traditional Chinese medicine. It is a rare orchid in the Orchidaceae with very short reproductive cycle as compared to others with life span of more than 3 years. Therefore, it can be used as a model to study various phenomena in orchids in a short time, especially the medicinal importance. This is the first report on the genetic regulation of bibenzyls and flavonoids in *A. graminifolia*. In addition to transcriptome dissection, the concentrations of bibenzyl, flavonoid, and phenylpropanoid were ascertained, showing the enormous medicinal importance of bamboo orchid.

Orchids are a rich source of important phytochemicals. Various active compounds, such as dendrobine, gigantol, nobiline, and moscatilin, have been found in the leaves and stems of *Dendrobium nobile* (Suzuki et al., 1973; Miyazawa et al., 1997; Zhao et al., 2001). The leaf extracts of *Coelogyne stricta* contained alkaloids, terpenoids, and phenols (Minh et al., 2016). The *Dendrobium pandurantum* is a rich source of phenols, alkaloids, tannins, flavonoids, and triterpenoids (Johnson and Janakiraman, 2013). Different parts of *Cymbidium aloifolium* (seeds, roots, leaves, and capsules) contained alkaloids, phenols, tannins, and cardiac glycosides (Shubha and Chowdappa, 2016). Other orchid species, such as *Monodora tenuifolia* (Ezenwali et al., 2010), *Eria pseudoclavicaulis* (Moin et al., 2012), *Rhynchostylis retusa* (Bhattacharjee and Islam, 2015), and *Vanda tessellate* (Bhattacharjee et al., 2015), have been shown to contain phytochemicals, such as alkaloids, phenols, tannins, coumarins, terpenoids, and flavonoids. Recent studies detected alkaloids and stilbenoids from the aerial parts of *D. officinale* and *A. graminifolia*, respectively (Auberon et al., 2016; Chen et al., 2019). High concentrations of phenylpropanoid, flavone, and bibenzyl were found in the aerial parts as well as roots of *A. graminifolia* (Figure 5D).

Cytochrome 450 is a vital regulator of secondary metabolites biosynthesis, such as bibenzyl (Adejobi et al., 2021). CYP84A1 (39318) is a critical enzyme in the phenylpropanoid biosynthetic pathway (Anderson et al., 2015). It was specifically expressed in the leaf both in qRT-PCR and transcriptome data (Figure 5E). However, CYP73A4 (41477) was expressed in the root, suggesting multiple accumulation points for bibenzyls in *A. graminifolia*. UDP-glycosyltransferases (UGTs) catalyze the transfer of glycosyl groups to acceptors, such as secondary metabolites and hormones (Bowles et al., 2005). High expression of UGT73C3 (38276) and UGT88B1 (34392) was observed in flower and leaf, respectively (Figure 5E). Regulators of flavonoids biosynthesis pathway (ANS, PAL, FLS, and 4CCL7) were highly expressed in leaf, suggesting leaf as the potent source of flavonoids other than stem (Figures 5D,E). Most of the genes important in the regulation of bibenzyls and flavonoids, presented in this study, have been identified in orchids and model crops (Supplementary Table 4). Sesquiterpenes are usually the product of farnesyl diphosphate (FPP) regulated by MVA and MEP pathways (Schwab and WüSt, 2015). Recently, several genes have been identified in FPP biosynthesis pathway (Chen et al., 2019; Adejobi et al., 2021). Most of the genes functioning in the initial biosynthesis stages of sesquiterpenes, such as DXR, DXS, MK, PMK, HMGS, HDR, and MDS, were present in our data. This signifies the involvement of these genes in the initial biosynthesis of bibenzyl in *A. graminifolia*. Genetic modification of their expression can boost bibenzyl content through *A. graminifolia*. Moreover, the CYP450 genes, such as CYP84A1 and CYP73A4, may be the critical genes regulating bibenzyl contents along with BIBSY212.

Orchids are useful pharmaceutical plants possessing anti-inflammatory, diuretic, anti-rheumatic, antiviral, neuroprotective, anti-carcinogenic, relaxation, anticonvulsive, antitumor, wound healing, hypoglycemic, anti-aging, antimicrobial, antioxidant, antibacterial, and anti-diarrheal uses (Ghanaksh and Kaushik, 1999; Moin et al., 2012; Islam et al., 2013; Pant, 2013; Rokaya et al., 2014; Bhattacharjee et al., 2015; Dalar et al., 2015). Although many studies have reported the medicinal components and uses of orchids, this study exploits the plant parts of an easily available orchid with large size. Large sized plants of *A. graminifolia* with profound vegetative growth and short reproductive cycle can generate enough plant waste even after the production of commercial flowers, as compared with any other orchid species. While discarding the orchid waste may cause environmental and health issues (Johnson et al., 1999; Srivirojana et al., 2005), it can be effectively used to obtain precious medicinal ingredients, such as bibenzyls. We found phenylpropanoid, flavone, and bibenzyl in multiple plant parts, such as stem, leaf, root, and flower (Figure 5D). This suggests whole plant of *A. graminifolia* is important to extract bioactive compounds for medicinal purposes than other orchid species. The short life cycle and accumulation of bioactive ingredients in multiple plant parts make bamboo orchid a favorite plant to study the efficacy of orchids to obtain pharmaceutical products.

## Conclusion

The bamboo orchid (*Arundina graminifolia*) is a special representative of the Orchidaceae family due to its short vegetative phase and continuous flowering pattern. Its medicinal importance is yet to be revealed at molecular levels. It grows big with long stems and large leaves, as compared with other orchids, and therefore, produces large waste after cut flower selection. This waste can be a valuable source to obtain different ingredients for drug development. Bibenzyl is a useful medicinal ingredient with numerous health benefits. Therefore, this first report discusses the molecular regulation of bibenzyls, flavonoids, and phenylpropanoids along with their multiple accumulation points in the plant body. The candidate genes included BIBSY212, CYP84A1, CYP73A4, 4CLL7, UGT88B1, UGT73C3, ANS, PAL, FLS, and CHS8, which play important role in the biosynthesis of bibenzyl, and flavonoids in *A. graminifolia*. Prominent concentrations of bibenzyl, phenylpropanoid, and flavone were found in the major plant parts, such as leaf, root, stem, flower, and fruit, suggesting multiple accumulation sites of phytochemicals in bamboo orchid. These interesting outcomes, therefore, broaden our current understanding on the medicinal efficacy of orchids.

## Data Availability Statement

The transcriptome data is deposited on the NCBI GeneBank with accession number: PRJNA844531.

## Author Contributions

SA: conceptualization, software, and writing-original draft. JG: data curation, formal analysis, and investigation. YW: visualization and investigation. CL: data curation and formal analysis. GZ: supervision, conceptualization, and funding acquisition. FY: supervision, conceptualization, funding acquisition, and writing-reviewing and editing. All authors contributed to the article and approved the submitted version.

## Funding

This research was funded by the Laboratory for Lingnan Modern Agriculture Project (NZ2021010), the Natural Science Foundation of Guangdong province (2017A030312004), grants from the National Key R&D Program (2018YFD1000400 and 2019YFD1001003), the Guangzhou Science and Technology Project (201707010307), the Innovation Team of Modern Agricultural Industry Technology System in Guangdong Province (2021KJ121), and the Guangdong Academy of Agricultural Sciences Discipline Team Construction Project (202127 TD, BZ202006, and R2020 PY-JX018).

## Conflict of Interest

The authors declare that the research was conducted in the absence of any commercial or financial relationships that could be construed as a potential conflict of interest.

## Publisher's Note

All claims expressed in this article are solely those of the authors and do not necessarily represent those of their affiliated organizations, or those of the publisher, the editors and the reviewers. Any product that may be evaluated in this article, or claim that may be made by its manufacturer, is not guaranteed or endorsed by the publisher.
